# Corrosion Properties of Cold-Sprayed Cr_3_C_2_-25(Ni20Cr) Coatings After Heat Treatment

**DOI:** 10.3390/ma17246289

**Published:** 2024-12-23

**Authors:** Mieczysław Scendo, Wojciech Żórawski

**Affiliations:** 1 Institute of Chemistry, Jan Kochanowski University in Kielce, Uniwersytecka 7, 25-406 Kielce, Poland; 2 Faculty of Mechatronics and Mechanical Engineering, Kielce University of Technology, Tysiąclecia Państwa Polskiego 7, 25-314 Kielce, Poland; ktrwz@tu.kielce.pl

**Keywords:** cold spray, Al7075 substrate, cermet coating, heat treatment, corrosion resistance, acidic chloride solution

## Abstract

The corrosion resistance of a Cr_3_C_2_-25(Ni20Cr) cermet coating applied to an Al7075 substrate (Cr_3_C_2_-25(Ni20Cr)/Al7075) was investigated. The coating was produced using a cold spraying (CS) method. The main aim of the research was to determine the effect of heat treatment on the properties of cermet coatings on the Al7075 substrate. The mechanical properties of the Cr_3_C_2_-25(Ni20Cr)/Al7075 composite were assessed through microhardness (HV) measurements. The surface morphology and microstructure of the specimens were examined using a scanning electron microscope (SEM). Electrochemical testing in an acidic chloride solution was employed to evaluate the corrosion behavior of the materials. The cermet coating effectively protected the Al7075 substrate from the aggressive corrosive environment. Heat treatment homogenized the structure of the cermet coating, eliminating microcracks and pores on the Cr_3_C_2_-25(Ni20Cr)/Al7075 surface. Notably, annealing at 300 °C in air significantly enhanced the corrosion resistance of the cermet coating. The corrosion rate was reduced by more than five times compared to the non-heat-treated Cr_3_C_2_-25(Ni20Cr)/Al7075 coating.

## 1. Introduction

A practical approach to protecting metals is by creating physical barriers to prevent exposure to elements like water, oxygen, and hydrogen. Metallic coatings and other types of protective layers are regarded as highly effective in this role. Among the various methods available, thermal spray processes including detonation-gun (DG), plasma, high-velocity air-fuel (HVAF), and high-velocity oxy-fuel (HVOF) spraying are commonly used to produce cermet coatings [1]. In the thermal spraying of fine Cr_3_C_2_–NiCr powders, chemical degradation of chromium carbides within the feedstock powder can occur, along with the dissolution of carbide phases into the NiCr matrix, forming phases such as Cr_7_C_3_ or Cr_24_C_6_ [2]. Consequently, using the cold spray (CS) method for Cr_3_C_2_–NiCr coatings can significantly minimize the decomposition effects often observed in fine Cr_3_C_2_–NiCr powders.

Cold spray is an emerging technology primarily used to produce and repair metal coatings, enhancing mechanical properties and improving corrosion resistance in various metal components. During the cold spraying process, metallic particles (ranging from 5 µm to 50 µm in diameter) are propelled at supersonic speeds through an inert gas flow in a de Laval nozzle, impacting the substrate to form metallic coatings. These particles collide with the substrate at velocities between 300 and 1200 m/s. As a result, the temperature of the process gas remains sufficiently low to prevent melting of the spray material [3,4,5]. If the velocity of the metal particles falls below a critical threshold, the substrate may experience damage due to abrasion. Notably, increasing the powder feed rate within the nozzle decreases the velocity of the deposited particles. This reduction occurs due to interactions between the gas and particles as they exit the nozzle [6,7,8].

Aluminum-zinc-magnesium (Al7075) alloys exhibit a stronger response to heat treatment compared to binary aluminum–zinc alloys, resulting in higher strength. However, the addition of zinc and magnesium reduces corrosion resistance, making it necessary to protect these alloys (such as Al7075) with corrosion-resistant metallic coatings. Titanium or nickel coatings are commonly applied for this purpose [9,10]. Traditional Cr_3_C_2_–NiCr coatings are widely used to add carbide cermet layers to industrial equipment, providing excellent resistance to wear, erosion, thermal shock, and stability at high temperatures in thermal spray applications. The effectiveness of these coatings relies heavily on factors such as deposition methods (parameters) and the coatings’ microstructure [11,12]. Moreover, chromium carbide particles in these coatings are dispersed in a nickel–chromium alloy matrix, with the Cr_3_C_2_–NiCr system specifically designed for applications requiring resistance to corrosion and wear.

The combination of ceramic and metal phases in Cr_3_C_2_–NiCr coatings contributes to achieving higher fracture strength. Notably, Cr_3_C_2_–NiCr coatings are known for their high hardness [13], which is directly linked to higher particle velocities and increased coating densities when applied to a substrate at ambient temperature. Corrosion resistance in cermet coatings is also influenced by surface roughness; a rougher surface leads to greater corrosion due to the increased surface area exposed [14]. Cr_3_C_2_–NiCr coatings are suitable for use in corrosive environments at service temperatures between 800 °C and 900 °C, making them ideal as protective coatings in high-temperature, corrosive settings. Various techniques, including heat treatment, sealing, and laser remelting, are employed to reduce defects in cermet coatings [15]. However, according to the literature, heat treatment has been relatively underutilized. Therefore, laboratory tests were conducted to explore the impact of heat remelting on enhancing the mechanical and anti-corrosion properties of cermet coatings on aluminum alloy substrates.

This study examined the effect of the heat remelting process on the corrosion resistance of the Cr_3_C_2_-25(Ni20Cr) cermet coatings applied to an Al7075 substrate. The coatings were produced using the cold spray method. To improve its properties, the coatings were annealed at 100 °C, 300 °C, and 500 °C for 24 h in air. Corrosion testing in an acidic chloride solution (1.2 M Cl^−^) was conducted through electrochemical techniques. Additionally, supplementary methods were employed to expand the scope of the research.

## 2. Experimental Details

The chemical composition of the Al7075 alloy is as follows by weight: 5.6% Zn, 2.5% Mg, 1.6% Cu, and 0.22% Cr, with minor admixtures (Mn, Fe, and Si) making up less than 0.50%, with the remainder being aluminum. As feedstock material, fine, irregular, and broken particles of the Cr_3_C_2_-25(Ni20Cr) (Diamalloy 3004, Oerlikon Metco Inc., Westbury, NY, USA) were used. This material consists of a mixture of Cr_3_C_2_ and Ni_2_OCr powders in a weight ratio of 75% to 25%.

**Figure 1:**
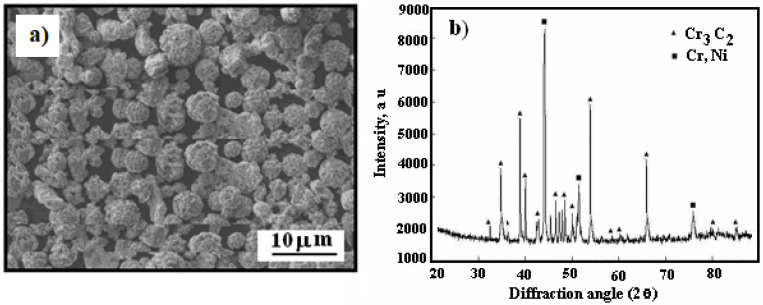
SEM micrograph: (**a**) Cr_3_C_2_-25(Ni20Cr) powder morphology, (**b**) X-ray diffraction pattern of powder.

Figure 1 presents a scanning electron microscopy (SEM) image showing the morphology of the powder, alongside an X-ray diffraction pattern of the Cr_3_C_2_-25(Ni20Cr) powder.

The Cr_3_C_2_ powder particles exhibit an irregular shape, whereas the Ni20Cr particles are aspherical, as shown in Figure 1a,b presents the X-ray diffraction pattern of the utilized powder. To reduce agglomeration effects, the powder was preheated to 110 °C in a convection oven for 1 h before being loaded into the feeder system.

The Cr_3_C_2_-25(Ni20Cr) cermet coating was applied to the Al7075 substrate (Cr_3_C_2_–25(Ni20Cr)/Al7075) using the Impact Innovations 5/8 cold spraying system equipped with a Fanuc M-20iA robot (Fanuc Robotics Ltd., Oshino, Japan). The cermet coatings were produced under the following conditions: nitrogen pressure at 40 bar, nitrogen preheating to 600 °C, a spraying distance of 60 mm, a traverse speed of 40 mm/s, a step size of 2 mm between each of the 10 passes, and four coating layers. The Al7075 substrate surface was prepared by blasting with corundum particles sized between 600 and 710 μm (size 30). The substrate specimen measured 310 × 110 × 5 mm^3^, and the resulting coating thickness ranged from 152 μm to 158 μm. Test samples were prepared in cuboid shapes with dimensions of 30 × 10 × 5 mm^3^.

**Figure 2:**
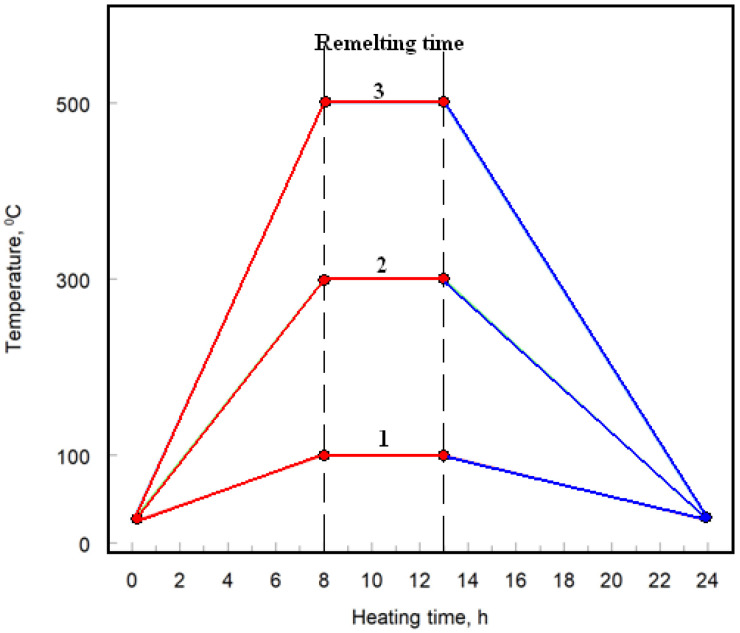
Heat treatment diagram of cermet coatings: 1, 2, and 3 heating programs.

Post-coating, the samples underwent heat treatment in an electric chamber furnace (CZYLOK, Jastrzebie Zdroj, Poland, model FCF 2.5 HM). The heat treatments were conducted in air at temperatures of 100 °C, 300 °C, and 500 °C, below the aluminum melting point of 660 °C. Each Cr_3_C_2_–25(Ni20Cr)/Al7075 cermet coated sample was heat treated for 24 h, with an additional remelting process applied to the surface for 5 h, as illustrated in Figure 2.

Additionally, Table 1 provides a detailed list of other heat treatment parameters for the cermet coatings, specifically for Cr_3_C_2_–25(Ni20Cr) at 100 °C, 300 °C, and 500 °C.

The surface morphology and microstructure were examined using a JSM-5400 scanning electron microscope (SEM) from Joel (Tokyo, Japan). The chemical composition was measured by energy dispersive spectrometer (EDS) (Joel, Tokyo, Japan). The X-ray diffraction (XRD) was employed to analyze the phase composition of the cold-sprayed coatings, utilizing a Bruker D8 Discover diffractometer (Bruker Ltd., Malvern, UK).

The microhardness was measured using the Vickers hardness (HV) method on an INNOVATEST Falcon 500 hardness tester (Maastricht, The Netherlands). A diamond pyramid indenter was applied with loads ranging from 0.02 N to 20 N, resulting in an indentation depth of approximately 3 μm.

To prepare the solutions, FLUKA (Alchem, Toruń, Polska) analytical grade sodium chloride and POCH (Pol-Aura, Morąg, Poland) analytical grade hydrochloric acid were used. The Cl^−^ ion concentration was set to 1.2 M, with a pH of 1.5. The electrolyte was used without deoxygenation.

The working electrode, made from Al7075 alloy coated with a cermet layer, had a geometric surface area of 1.0 cm^2^.

The saturated calomel electrode (SCE(KCl)) served as the reference electrode.

The counter electrode was made of a platinum mesh (99.9% Pt) and had a surface area of 9 cm^2^.

All electrochemical measurements were taken using a PGSTAT 128N potentiostat/galvanostat (AutoLab, Amsterdam, The Netherlands) with NOVA 1.7 software. Potentiodynamic polarization (LSV) curves were used to determine the electrochemical corrosion parameters of the tested materials.

The corrosion parameters for each material were determined as average values from three measurements.

The chronoamperometric (ChA) curves were obtained at specific potential values selected based on the LSV curves. These potentials for the working electrode were carefully chosen to capture changes in current density at characteristic points on the LSV curves. For each tested material, three potentials were selected. This approach enables the assessment of the anti-corrosive properties of the cermet coatings on the Al7075 substrate.

The measurements were conducted at a controlled temperature of 25 ± 0.5 °C, maintained by an air thermostat.

## 3. Results and Discussion

### 3.1. Surface Morphology

**Figure 3:**
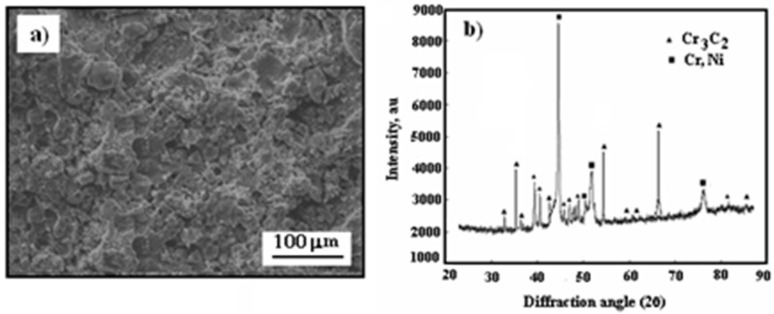
Surface morphology of cold-sprayed cermet coating on the Al7075 substrate: (**a**) Cr_3_C_2_–25(Ni20Cr)/Al7075, (**b**) X-ray diffraction pattern of as-sprayed coating.

Figure 3 displays the surface morphology and X-ray diffraction pattern of the cermet coating on the Al7075 substrate in its as-sprayed state.

The surface of the cold-sprayed cermet coating on the Al7075 substrate appears compact yet uneven and undulating (Figure 3a). As shown in Figure 3b, the primary diffraction peaks correspond to Cr_3_C_2_ and (Cr, Ni) phases, resembling the X-ray diffraction pattern of Cr_3_C_2_–25(Ni20Cr) powder (Figure 1b).

**Figure 4:**
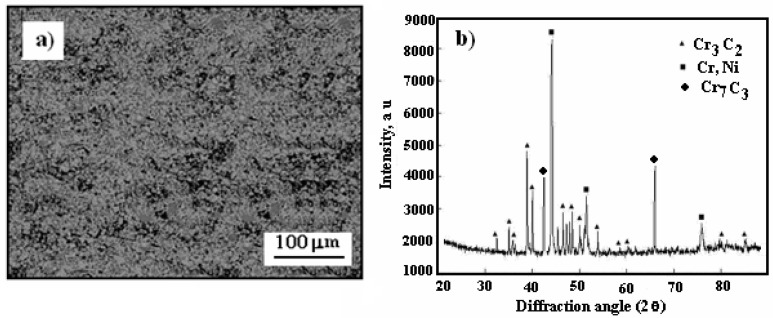
Surface morphology of cold-sprayed cermet coating on the Al7075 substrate after heat treatment at 300 °C: (**a**) Cr_3_C_2_–25(Ni20Cr)/Al7075, (**b**) X-ray diffraction pattern of coating.

In Figure 4, the surface morphology of the cold-sprayed cermet coating on the Al7075 substrate after heat treatment at 300 °C, along with its X-ray diffraction pattern, is presented.

It is noteworthy that the surface of the Cr_3_C_2_–25(Ni20Cr) coating on the Al7075 substrate became smoother following heat treatment at 300 °C in a hot air atmosphere (Figure 4a). However, the post-heat treatment surface remains compact and tight, which should effectively shield the substrate from corrosive environments. Additionally, a new diffraction peak for the Cr_7_C_3_ phase was detected at 2θ = 44.17° on the cermet coating surface (Figure 4b), suggesting the formation of this new phase through the restructuring and partial decarburization of Cr_3_C_2_ as per the following reaction:

7Cr_3_C_2_ → 3Cr_7_C_3_ + 5C (1)

**Figure 5:**
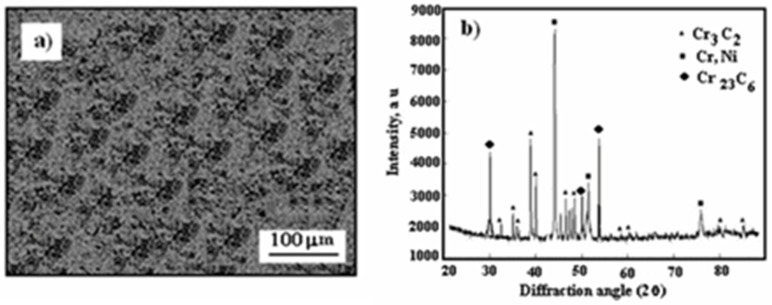
Surface morphology of cold-sprayed cermet coating on the Al7075 substrate after heat treatment at 500 °C: (**a**) Cr_3_C_2_–25(Ni20Cr)/Al7075, (**b**) X-ray diffraction pattern of coating.

Figure 5 illustrates the surface morphology and X-ray diffraction pattern of the cold-sprayed cermet coating on the Al7075 substrate after heat treatment at 500 °C.

The high-temperature heat treatment at 500 °C resulted in noticeable changes in the surface structure of the cermet coating, as shown in Figure 5a. Numerous depressions formed on the Cr_3_C_2_–25(Ni20Cr)/Al7075 surface, which considerably compromised the coating’s integrity and likely reduced its anti-corrosion effectiveness. However, under these elevated temperature conditions, a new phase appeared on the surface of the Cr_3_C_2_–25(Ni20Cr)/Al7075 coating, as observed in Figure 5b, which likely formed due to the following reaction:

23Cr_7_C_3_ → 7Cr_23_C_6_ + 27Cr (2)

In the hot air atmosphere, further decarburization occurs on the Cr_3_C_2_–25(Ni20Cr)/Al7075 surface:

23Cr_3_C_2_ → 3Cr_23_C_6_ + 28C (3)

C + O_2_ → CO_2_ (4)

2C + O_2_ → 2CO (5)

As a result, metastable carbides (Cr_7_C_3_ and Cr_23_C_6_) may form at the interface between Cr_3_C_2_ and Ni-Cr phases. Consequently, under high-temperature conditions (500 °C), structural transformations are observed on the Cr_3_C_2_–25(Ni20Cr)/Al7075 surface due to reactions (1)–(3), leading to a reduction in the anti-corrosion effectiveness of the cermet coating. Additionally, carbon is released as CO or CO_2_ gases through reactions (4) and (5). Similar conclusions were reached by [16], though this issue will be explored in further detail later in this study.

### 3.2. Microstructure of Cermet Coatings

**Figure 6:**
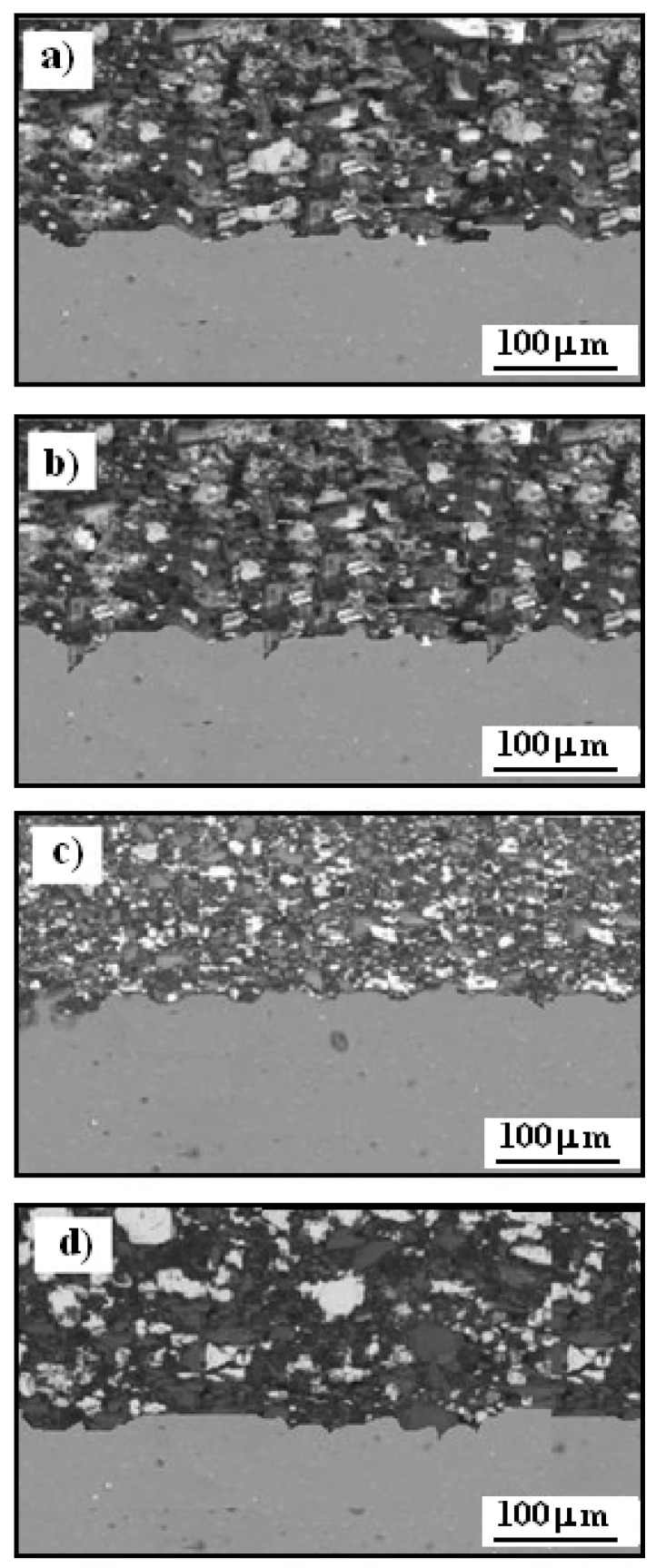
SEM of cross-section of Cr_3_C_2_-25(Ni20Cr)Al7075 cermet coatings: (**a**) before and after heat treatment at: (**b**) 100 °C, (**c**) 300 °C, and (**d**) 500 °C.

Figure 6 presents the scanning electron microscopy (SEM) cross-sectional microstructure of Cr_3_C_2_–25(Ni20Cr)/Al7075 cermet coatings, both before and after heat treatment.

The coatings were produced using the cold spraying method, resulting in a strong bond with the substrate, with no visible cracks and densely packed carbide particles. The microstructure shows distinct contrast zones—dark gray, medium gray, and light gray—highlighted by the varying shades in the coating. The dark gray regions in the Cr_3_C_2_–25(Ni20Cr)/Al7075 coatings consist of NiCr and carbon, and these areas are identified as carbide zones. The light gray regions are primarily associated with chromium carbides, specifically Cr_3_C_2_ and Cr_7_C_3_, while the medium gray phase represents the more complex chromium carbide, Cr_23_C_6_. Figure 6a shows the cross-section of the Cr_3_C_2_-25(Ni20Cr) cermet coating on the Al7075 substrate prior to heat treatment, while Figure 6b illustrates the cross-section after heat treatment at 100 °C. No substantial structural changes were observed on the Cr_3_C_2_-25(Ni20Cr) coating at this temperature. However, after heat treatment at 300 °C, noticeable structural modifications occurred, as shown in Figure 6c. The fine structure of chromium carbides became more distinct in the Cr_3_C_2_-25(Ni20Cr) coating cross-section, with heat treatment reducing microcracks and pores on the Cr_3_C_2_-25(Ni20Cr)/Al7075 surface. When the temperature was raised to 500 °C, the fine-crystalline structure of chromium carbides was no longer visible, as illustrated in Figure 6d. Additionally, elemental carbon appeared within the coating structure, which likely contributed to a significant reduction in the anti-corrosion properties of the Cr_3_C_2_-25(Ni20Cr) coating.

**Figure 7:**
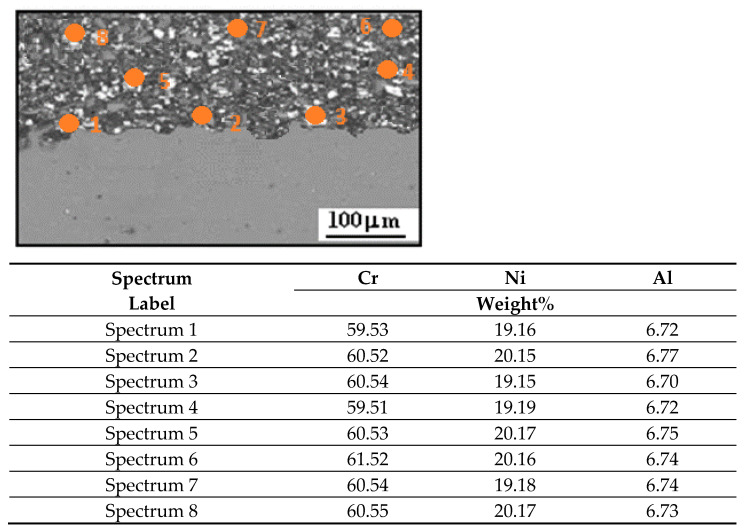
SEM image cross-section of the Cr_3_C_2_-25(Ni20Cr)/Al7075 cermet coating after heat treatment at 300 °C and the results of point X-ray microanalysis of the chemical composition of the tested material.

Figure 7 depicts the SEM/EDS image of a cross-section of the Cr_3_C_2_-25(Ni20Cr)/Al7075 cermet coating after heat treatment at 300 °C and the results of a point X-ray microanalysis of the chemical composition of the tested material.

The average metal content in the Cr_3_C_2_-25(Ni20Cr) coatings was 60.41%, 19.67%, and 6.67% for the elements Cr, Ni, and Al, respectively. The quantitative distribution of the elements along the cross-section of the coatings is the same. Thus, as a result of heat treatment, a homogeneous distribution of Cr, Ni, and Al was obtained along the cross-section of the coating.

### 3.3. Microhardness

Table 2 illustrates the impact of heat treatment on the microhardness (HV10) of Cr_3_C_2_–25(Ni20Cr)/Al7075 cermet coatings, comparing values before and after heat treatment.

The measurement results in Table 2 reveal significant variability in HV10 microhardness values due to the heterogeneous nature of the cold-sprayed Cr_3_C_2_-25(Ni20Cr) coating surface. This variability arises from particles with differing deformation levels, influenced by a broad particle size distribution and varying positions in the spray jet, resulting in indentation values that depend on particle size. Heat treatment was found to alter the surface microhardness of the cermet coating on the Al7075 substrate. At 300 °C, the HV10 microhardness of the Cr_3_C_2_-25(Ni20Cr)/Al7075 coating nearly doubled compared to the untreated coating. However, further increasing the heat treatment temperature to 500 °C led to a substantial decrease in microhardness by approximately 150 units on the HV10 scale compared to the coating treated at 300 °C (as shown in Table 2). This indicates that heat treatment affects the Cr_3_C_2_-25(Ni20Cr)/Al7075 surface structure by smoothing and hardening it, with the most pronounced effect observed at 300 °C. At lower heat treatment temperatures, such as 100 °C, the Cr_3_C_2_-25(Ni20Cr) coating experiences only superficial melting, leading to minimal structural and mechanical changes in the surface of the cermet coating.

### 3.4. Corrosion Test

**Figure 8:**
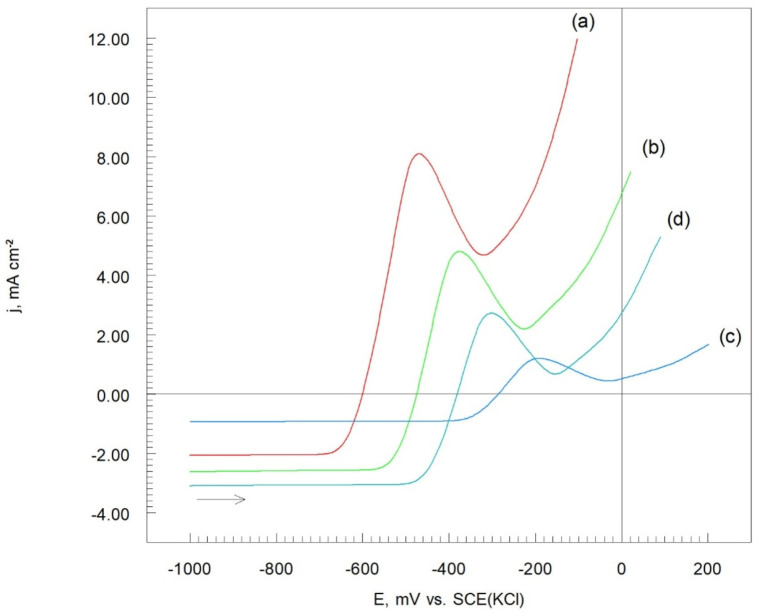
Potentiodynamic polarization curves of Cr_3_C_2_-25(Ni20Cr)/Al7075 cermet coatings: (**a**) before, and after heat treatment at (**b**) 100 °C, (**c**) 300 °C, and (**d**) 500 °C. Solution contained 1.2 M Cl^−^, pH 1.5, d*E*/d*t* 1 mV/s.

The corrosion resistances of the Cr_3_C_2_-25(Ni20Cr)/Al7075 cermet coatings were evaluated through electrochemical testing in an acidic chloride solution. Figure 8 displays the potentiodynamic polarization (LSV) curves for the Cr_3_C_2_-25(Ni20Cr)/Al7075, both before and after heat treatment.

Hydrogen depolarization occurs in the cathodic region of the potentiodynamic polarization curves. In the acidic corrosive environment, the cathodic branches of the LSV curves represent the simplified reduction of hydrogen ions [9]:

Me^0^ + nH^+^→Me^0^ + nH_2_ − me^−^ (6)

where Me means the Cr, Ni, and other metals.

Conversely, the anodic reaction proceeds as follows:

Me^0^ + 2H^+^ + O_2_→(MeO)_ads_ + H_2_O + me^−^ (7)

In this context, (MeO)_ads_ refers to oxides such as (Cr_2_O_3_)_ads_ and (NiO)_ads_. It was found that the anodic current density values vary based on the heat treatment temperature of the cermet coatings on the Al7075 substrate, as shown in Figure 8. Notably, the lowest anodic current density values were observed for the Cr_3_C_2_-25(Ni20Cr)/Al7075 coating heat treated at 300 °C (Figure 8c). When the heat treatment temperature was increased to 500 °C, the anodic current density also increased (Figure 8d). Therefore, the most effective anti-corrosion properties of the Cr_3_C_2_-25(Ni20Cr)/Al7075 coating were achieved with heat treatment at 300 °C in a hot air atmosphere.

Characteristic peaks were observed at −480 mV, −370 mV, −200 mV, and −305 mV vs. SCE(KCl) on the anodic segments of the potentiodynamic polarization curves for the Cr_3_C_2_-25(Ni20Cr)/Al7075 coatings (Figure 8). This suggests that the working electrode surface was primarily covered with a layer of (Cr_2_O_3_)_ads_ and (NiO)_ads_ oxides, indicating that the Cr_3_C_2_-25(Ni20Cr)/Al7075 coating was passivated under the test conditions, with the oxides adhering well to the surface. Additionally, it appears that this adsorbed oxide layer can be further stabilized by the adsorption of Cl^−^ ions:

(MeO)_ads_ + Cl^−^ + H^+^ → (MeClOH)_ads_ (8)

The adsorption layer (MeClOH)_ads_ dissolves according to the following chemical reaction:

(MeClOH)_ads_ + H^+^ → Me^n+^ + Cl^−^ + H_2_O (9)

This dissolution leads to a further sharp increase in anodic current density as the electrode surface undergoes oxidation (Figure 8).

#### 3.4.1. Corrosion Electrochemical Parameters

**Figure 9:**
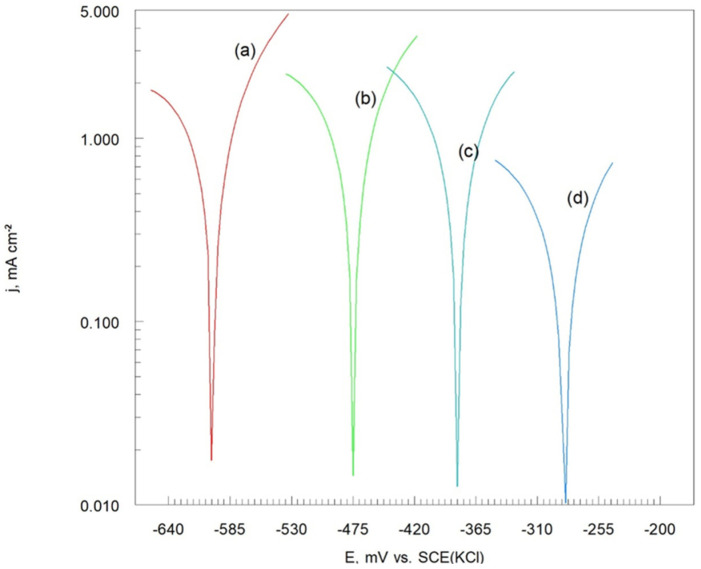
Potentiodynamic polarization curves on a semi-logarithmic (Tafel scale) of Cr_3_C_2_-25(Ni20Cr)/Al7075 cermet coatings: (**a**) before, and after heat treatment at (**b**) 100 °C, (**c**) 300 °C, and (**d**) 500 °C. Solution contained 1.2 M Cl^−^, pH 1.5.

The potentiodynamic polarization curves of the Cr_3_C_2_-25(Ni20Cr)/Al7075 cermet coatings were analyzed to determine the electrochemical corrosion parameters of the tested coatings, as shown in Figure 9.

It was observed that the corrosion potential (E_corr_) values of the tested materials shift towards more positive values with increasing heat treatment temperature, as shown in Table 3. This indicates that heat treatment enhances the corrosion resistance of the cermet coatings on the Al7075 substrate. However, once the temperature reaches 500 °C, the corrosion potential shifts to more negative values, suggesting a loss of anti-corrosion properties in the Cr_3_C_2_–25(Ni20Cr)/Al7075 coating.

At the same time, the lowest corrosion current density j_corr_ = 1.95 mA/cm^2^ observed for the Cr_3_C_2_–25(Ni20Cr) coating treated at 300 °C suggests optimal anti-corrosion performance at this temperature (Table 3). Notably, the slopes of the Tafel polarization curves, specifically (−b_c_) and (b_a_), remain relatively unchanged with increasing heat treatment temperatures (Table 3). This stability in slope values indicates that the corrosion mechanism of the Cr_3_C_2_–25(Ni20Cr)/Al7075 cermet coatings is not significantly affected by surface heat treatment.

#### 3.4.2. Polarization Resistance and Corrosion Rate

The polarization resistance (R_p_) of the Cr_3_C_2_–25(Ni20Cr)/Al7075 coatings were calculated based on the slope values (Table 3) derived from the Tafel potentiodynamic polarization curves (Figure 9). Additionally, for the reaction:

Cr^0^ − 3e^−^ → Cr^3+^ (10)

The corrosion rate (CR) of the materials was calculated using the following equation:

CR (mm/y) = 7.93 × 10^−3^ j_corr_ (11)

The R_p_ and CR values for the cermet coatings on the Al7075 alloy are summarized in Table 4.

The highest polarization resistance (R_p_) value, 3817 kΩ cm^2^, was observed for the Cr_3_C_2_–25(Ni20Cr)/Al7075 coating that underwent heat treatment at 300 °C. This high R_p_ value (Table 4) indicates a significant impediment to mass and electric charge exchange between the working electrode and the solution. Additionally, the corrosion rate (CR) for the Cr_3_C_2_–25(Ni20Cr) coating treated at 300 °C was the lowest, at 0.018 mm/y. These findings confirm the earlier conclusion that the Cr_3_C_2_–25(Ni20Cr) coating on the Al7075 substrate exhibits optimal anti-corrosion properties when heat treated in an air atmosphere at 300 °C.

#### 3.4.3. Chronoamperometric Measurements

**Figure 10:**
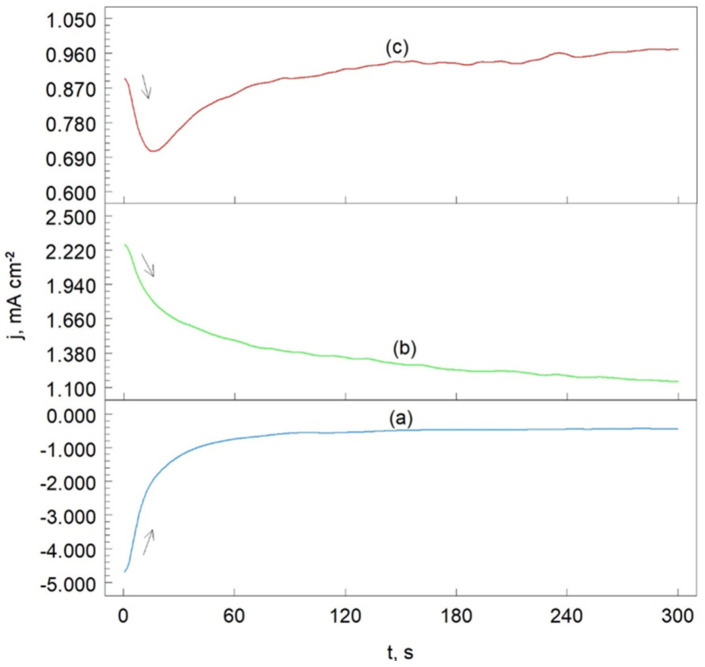
Chronoamperometric curves obtained after heat treatment at 300 °C of Cr_3_C_2_-25(Ni20Cr)/Al7075 cermet coating. The potential values were as follows: (**a**) −800 mV, (**b**) −200 mV, and (**c**) +100 mV. Solutions contained 1.2 M Cl^−^, pH 1.5.

Chronoamperometry (ChA) is the study of current response over time at a specifically chosen potential. Figure 10 presents the chronoamperometric curves for the Cr_3_C_2_-25(Ni20Cr)/Al7075 cermet coating after heat treatment at 300 °C in an acidic chloride solution. Similar ChA curves were also obtained for the Cr_3_C_2_-25(Ni20Cr)/Al7075 coatings subjected to heat treatments at 100 °C and 500 °C.

The working electrode potentials were chosen based on the potentiodynamic polarization curve (Figure 8, curve (c)). At a potential of −800 mV vs. SCE(KCl), the reduction in H^+^ ions (reaction (6)) occurs on the electrode surface (Figure 10, curve (a)). Initially, the cathodic current density decreases and then stabilizes at approximately 0.8 mA cm^2^, indicating that the H^+^ reduction process is stable under these experimental conditions.

At −200 mV vs. SCE(KCl) (Figure 10, curve (b)), oxidation of the Cr_3_C_2_-25(Ni20Cr) cermet coating is observed (reaction (7)). Here, the anodic current density systematically decreases, as the oxide layer on the Cr_3_C_2_-25(Ni20Cr)/Al7075 surface becomes sealed through the adsorption of (Cr_2_O_3_)_ads_, and (NiO)_ads_ oxides due to reaction (7). This adsorbed layer of chromium and nickel oxides effectively seals the Cr_3_C_2_-25(Ni20Cr)/Al7075 coating. In the chloride environment, the passive oxide layer can be further sealed through the adsorption of Cl^−^ ions, as described by the reaction (8).

For a more positive working electrode potential of +100 mV, the initial oxidation current density of the Cr_3_C_2_-25(Ni20Cr)/Al7075 surface decreases (up to 20 s), and then gradually increases with extended electrolysis time (Figure 10, curve (c)). The initial decrease in anodic current density results from the dissolution of the (MeClOH)_ads_ layer as described by reaction (9). This suggests that in the acidic chloride solution, the protective layer adsorbed on the electrode surface is gradually dissolved, leading to the onset of the corrosion process in the Cr_3_C_2_(Ni20Cr)Al7075 cermet coating.

### 3.5. Surface Morphology After Corrosion Test

**Figure 11:**
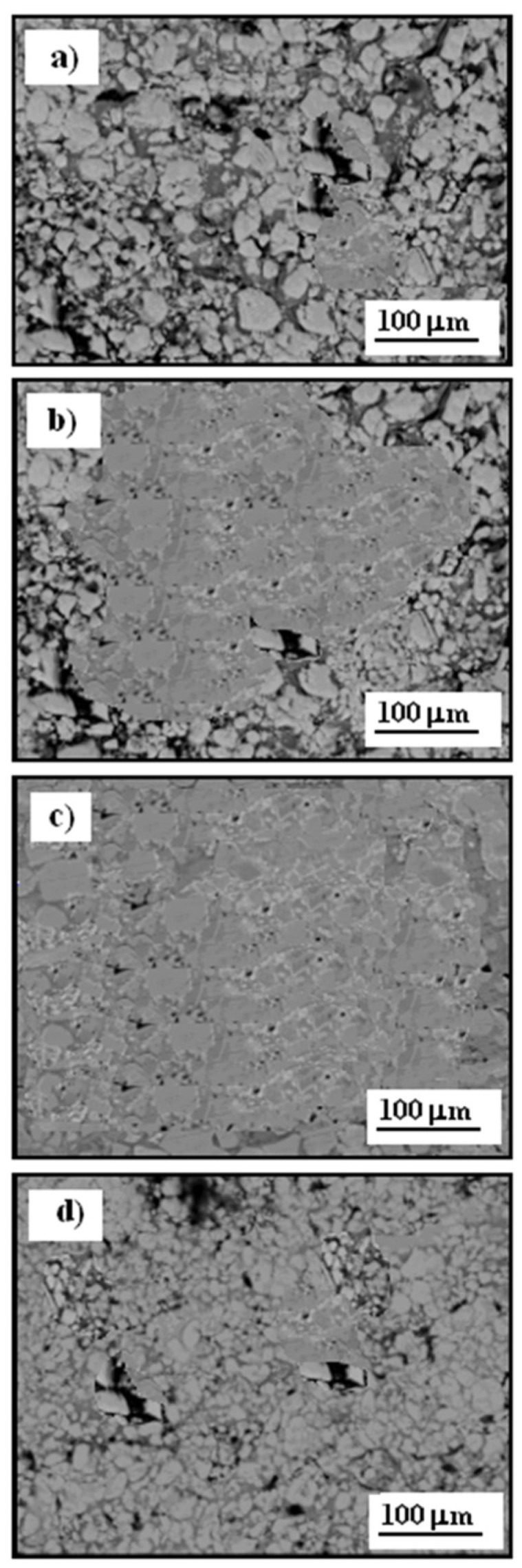
SEM of surface morphology of Cr_3_C_2_-25(Ni20Cr)/Al7075 cermet coatings after corrosion test: (**a**) before, and after heat treatment at (**b**) 100 °C, (**c**) 300 °C, and (**d**) 500 °C. Solutions contained 1.2 M Cl^−^, pH 1.5. Exposure time was 5 h.

Figure 11 presents scanning electron microscopy (SEM) images of the surface morphology of the Cr_3_C_2_-25(Ni20Cr)/Al7075 cermet coatings following a corrosion test in an acidic chloride solution for an exposure time of 5 h. The oxide layer on the test specimens was subsequently removed using diluted nitric acid, with an exposure time of approximately three minutes.

Figure 11a shows the surface of the Cr_3_C_2_-25(Ni20Cr) coating without heat treatment, where extensive surface damage is visible due to prolonged exposure to the electrolyte. Numerous deep pits formed as a result of the corrosion process, significantly diminishing the mechanical and esthetic qualities of the material. Slightly less corrosion damage is observed on the Cr_3_C_2_-25(Ni20Cr) surface after heat treatment at 100 °C, as shown in Figure 11b, indicating that low-temperature heat treatment does not substantially improve the coating’s anti-corrosion properties. The least corrosion damage is observed in Figure 11c, representing the Cr_3_C_2_-25(Ni20Cr)/Al7075 surface treated at 300 °C. In this case, the heat treatment effectively hardened and sealed the coating, which significantly slowed the corrosion process and enhanced the material’s resistance in the corrosive environment.

Conversely, after heat treatment at 500 °C, the Cr_3_C_2_-25(Ni20Cr)/Al7075 surface showed low resistance to the corrosive environment. Extended exposure led to the formation of numerous deep pits on the Cr_3_C_2_-25(Ni20Cr)/Al7075 surface due to electrochemical corrosion in the chloride environment, as shown in Figure 11d. Therefore, the tested cermet coating, when heat-treated at this higher temperature, does not effectively protect the Al7075 substrate from corrosive contact.

## 4. Conclusions

This paper presents research findings on the impact of heat treatment on the anti-corrosion properties of cold-sprayed Cr_3_C_2_-25(Ni20Cr) coatings on the Al7075 substrate in an acidic chloride solution. The results led to the following conclusions:

1. The heat treatment at 300 °C resulted in the most uniform and smooth structure for the cermet coating.
2. Annealing introduced new phases, specifically Cr_7_C_3_ and Cr_23_C_6_, which formed on the cermet surface due to the restructuring and partial decarburization of Cr_3_C_2_.
3. The highest microhardness value was achieved for the coating annealed at 300 °C.
4. The electrochemical corrosion mechanism of the cermet coatings involves multiple stages, with (Cr_2_O_3_)_ads_, and (NiO)_ads_ particles as the primary corrosion products. However, this oxide layer did not effectively shield the substrate from penetration of corrosive solution.
5. The Cr_3_C_2_-25(Ni20Cr)/Al7075 coating heat treated in an air atmosphere at 300 °C exhibited the highest polarization resistance and lowest corrosion rate, significantly reducing the exchange mass and electric charge between electrode and electrolyte solution.
6. Coatings annealed at 100 °C, and 500 °C suffered severe damage from electrochemical corrosion after exposure to an aggressive environment, indicating compromised resistance.

## Figures and Tables

**Table 1:** Parameters of heat treatment of cermet coatings.

Program Number	Heating Rate °C/h	Temperature °C	Cooling Rate °C/h
1	12.5	100	9.1
2	37.5	300	27.3
3	62.5	500	45.5

**Table 2:** Microhardness of Cr_3_C_2_–25(Ni20Cr)/Al7075 cermet coatings before, and after heat treatment.

Sample Name	Microhardness HV10
Cr_3_C_2_–25(Ni20Cr)	326 ± 4
Cr_3_C_2_–25(Ni20Cr)—100	478 ± 2
Cr_3_C_2_–25(Ni20Cr)—300	695 ± 1
Cr_3_C_2_–25(Ni20Cr)—500	549 ± 3

**Table 3:** Corrosion electrochemical parameters of Cr_3_C_2_–25(Ni20Cr)Al7075 cermet coatings before, and after heat treatment.

Sample Name	E_corr_ mV vs. SCE(KCl)	j_corr_ mA cm^−2^	−b_c_	b_a_
			mV dec^−1^	
Cr_3_C_2_–25(Ni20Cr)	−602	10.23	62	34
Cr_3_C_2_–25(Ni20Cr)—100	−475	7.94	48	34
Cr_3_C_2_–25(Ni20Cr)—300	−285	1.95	40	30
Cr_3_C_2_–25(Ni20Cr)—500	−379	4.79	32	28

**Table 4:** Polarization resistance and corrosion rate of Cr_3_C_2_–25(Ni20Cr)Al7075 cermet coatings before and after heat treatment.

Sample Name	R_p_ kΩ cm^2^	CR mm/y
Cr_3_C_2_–25(Ni20Cr)	932	0.081
Cr_3_C_2_–25(Ni20Cr)—100	1088	0.073
Cr_3_C_2_–25(Ni20Cr)—300	3817	0.018
Cr_3_C_2_–25(Ni20Cr)—500	1354	0.044

## Data Availability

The original contributions presented in this study are included in the article. Further inquiries can be directed to the corresponding author.
